# Nationwide surveillance of antimicrobial susceptibility of 509 rapidly growing mycobacteria strains isolated from clinical specimens in Japan

**DOI:** 10.1038/s41598-021-91757-4

**Published:** 2021-06-09

**Authors:** Keisuke Kamada, Atsushi Yoshida, Shigekazu Iguchi, Yuko Arai, Yutaka Uzawa, Satoshi Konno, Masahiro Shimojima, Ken Kikuchi

**Affiliations:** 1 Department of Infectious Diseases, Tokyo Women’s Medical University, 8-1 Kawada-Cho, Shinjuku-ku, Tokyo, 162-8666 Japan; 2 Department of Respiratory Medicine, Faculty of Medicine and Graduate School of Medicine, Hokkaido University, Kita 14-jo Nishi 5-chome, Kita-ku, Sapporo-shi, Hokkaido, 001-0014 Japan; 3 Department of Mycobacterium Reference and Research, The Research Institute of Tuberculosis, Japan Anti-Tuberculosis Association, Tokyo, 204-0022 Japan; 4 BML, Inc, Matoba 1361-1, Kawagoe-shi, Saitama, 350-1101 Japan

**Keywords:** Antimicrobial resistance, Clinical microbiology

## Abstract

This study aimed to identify effective treatments against rapidly growing mycobacteria (RGM) infections by investigating the minimum inhibitory concentrations (MIC) of 24 antimicrobial agents and their molecular mechanisms of resistance. In total, 509 clinical RGM isolates were identified by analyzing the sequences of three housekeeping genes (*hsp65*, *rpoB*, and *sodA*), and their susceptibilities to 24 antimicrobial agents were tested. We also performed sequencing analysis of antimicrobial resistance genes (*rrl*, *rrs*, *gyrA*, and *gyrB*). To identify *Mycobacteroides abscessus* group subspecies, we performed PCR-based typing and determined the sequevar of *erm*(41). We identified 15 RGM species, most of which were susceptible to amikacin and linezolid. Among these species, arbekacin and sitafloxacin had the lowest MIC among the same class of antimicrobials. The MIC of rifabutin for *M. abscessus* subsp. *abscessus* (MAB) was lower than that for *M. abscessus* subsp. *massiliense* (MMA). The proportion of MAB isolates with MIC ≤ 2 mg/L for rifabutin was significantly higher than that of MMA [MAB: 50/178 (28.1%) vs. MMA: 23/130 (17.7%); *p* = 0.041]. In summary, our study revealed the antimicrobial susceptibility profile of 15 RGM species isolated in Japan and indicated that arbekacin, sitafloxacin, and rifabutin may be possible therapeutic options for RGM infections.

## Introduction

Rapidly growing mycobacteria (RGM) infections constitute a serious public health concern worldwide, particularly in East Asia, and the proportion of RGM among nontuberculous mycobacteria (NTM) is high^1^. The prevalence of infections caused by the *Mycobacteroides abscessus* group (MAG), a major group of RGM, has increased in Japan^2^. Several mycobacterial species causing RGM infections have a natural resistance to several antimicrobials, rendering standard treatment regimens inefficient^3^. Several gene mutations related to drug susceptibility or resistance in RGM have been reported, including: *erm*(41) C28 sequevar, which is related to macrolide susceptibility^4^; *rrl*, which is associated with acquired resistance to macrolides^4^; *rrs*, which affects aminoglycoside resistance^5^; and *gyrA* and *gyrB*, which encode a quinolone resistance-determining region (QRDR) related to emerging quinolone resistance^6^.

Of these genes, the most important is *erm*(41), which is involved in the macrolide-induced resistance of MAG. When MAG is exposed to a macrolide, the *erm*(41) gene is expressed, and the product of this gene methylates the macrolide binding site on 23S rRNA^7^. This inhibits the action of the macrolide, resulting in drug resistance. To accurately evaluate this induced resistance, it is necessary to wait until the 14th day after the start of the drug susceptibility test^8^.

There are differences in the *erm*(41) gene sequence among MAG subspecies.

In *M. abscessus* subsp. *massiliense* (MMA), there is a deletion in the *erm*(41) gene, and its function is lost^9^. Therefore, MMA is macrolide-susceptible, whereas *M. abscessus* subsp. *abscessus* (MAB) and *M. abscessus* subsp. *bolletii* (MBO), which do not have this deletion, are often macrolide-induced resistant^9^. However, it is known that this macrolide-induced resistance is lost because of the T28C mutation in the *erm*(41) gene, even in the *erm*(41) gene without the deletion^4^.

Previous reports in the USA showed that there were 10 different sequevar types in the *erm*(41) gene, and that the sequevar types with C28 (type 2, 3, and 5) were macrolide-susceptible, whereas the other sequevar types with T28 (type 1, 4, 6, 7, 8, 9, and 10) were mostly macrolide-resistant^10^. Because differences in the sequence of the *erm*(41) gene are useful in predicting susceptibility to macrolides, the Clinical Laboratory Standards Institute (CLSI) recommends that the sequevar type of the *erm*(41) gene be evaluated in MAB^11^. However, in actual clinical practice in Japan, the *erm*(41) sequevar type is rarely determined, strictly due to the labor and cost required for testing, and these epidemiological data are unknown.

Susceptibility of RGM to antimicrobials remains controversial for multiple reasons. First, because of the high degree of phylogenetic similarity between different RGM, accurate species identification requires detailed genetic analysis. Previously reported large-scale antimicrobial susceptibility tests have not always identified RGM species with sufficient accuracy^12^. The minimum inhibitory concentration (MIC) breakpoints of only 11 antimicrobials are described in CLSI M24A-2^8^, and the MIC values of other antimicrobials that have been measured according to the CLSI method are not sufficiently evaluated. Second, recent reports demonstrate that susceptibility to macrolides correlates with the response rate^13, 14^, and that the use of azithromycin, imipenem, and amikacin is associated with good therapeutic results^15^ for pulmonary infections caused by MAG. However, the correlation between breakpoints proposed by CLSI and treatment outcomes remains unclear for most antimicrobials in many settings of RGM infection. Gathering information regarding the MIC values of antimicrobials that can be used as therapeutic options for treating infections caused by RGM species is essential. Third, epidemiological information related to the gene mutations involved in antimicrobial resistance is scarce.

Therefore, the current study aimed to determine the MIC of 24 antimicrobial agents for clinically isolated RGM and record relevant epidemiological and genetic information to identify potential therapeutic agents.

## Results

The details of 15 species that were identified are shown in Table 1. Eleven isolates, *M. abscessus* subsp. *abscessus* (MAB) (5), *M. abscessus* subsp. *massiliense* (MMA) (3), *M. chelonae* (2), and *M. senegalense* (1) grew poorly in the culture medium at five days after the start of susceptibility test, and thus we could not obtain MIC data for these isolates.

**Table 1:** Distribution of rapidly growing mycobacteria (RGM) species by specimen type.

Pathogens	Total	No. of isolates									Unkown
		LRS	NLRS total	NLRS							
				Skin, soft tissue	Abscess	CAPD-related	Blood	Otrrhea	Bone	Other	
MAG	321	280	31	8	6	4	4	6	2	1	10
subsp *abscessus* (MAB)	183	164	14	2	4	3	0	2	2	1	5
subsp *massiliense* (MMA)	133	111	17	6	2	1	4	4			5
subsp *bolletii* (MBO)	5	5	0								
*M. fortuitum*	85	67	18	9	3	3	1		1	1	
*M. chelonae*	57	37	19	12	2	1	2			2	1
*M. peregrinum*	11	11	0								
*M. mageritense*	10	1	8	3	1	2	1			1	1
*M. septicum*	5	4	1				1				
*M. mucogenicum*	4	1	2		1		1				1
*M. porcinum*	3	2	1		1						
*M. wolinskyi*	3	0	3		2		1				
*M. senegalense*	3	3	0								
*M. goodii*	2	1	1							1	
*M. iranicum*	2	1	1			1					
*M. canariasense*	1	0	1				1				
*M. immunogenum*	1	0	1		1						
*M. sphagni*	1	1	0								
Total	509	409	87	32	17	11	12	6	3	6	13

*PD* Peritoneal dialysis, *LRS* lower respiratory specimens, *NLRS* non-lower respiratory specimens.

### Characteristics of antimicrobial susceptibilities of RGM species

Other than MMA, which was susceptible to both amikacin and clarithromycin, MAB and *M. abscessus* subsp. *bolletii* (MBO) were susceptible to only amikacin (Table 2). Although *M. fortuitum* was resistant to macrolides, it was susceptible to amikacin, imipenem, fluoroquinolones, and trimethoprim/sulfamethoxazole (Table 3). Only three isolates were not susceptible to fluoroquinolones. Most *M. chelonae* isolates were susceptible to clarithromycin. However, the proportion of isolates intermediate and resistant to aminoglycosides, imipenem, cefoxitin, and fluoroquinolones was high. Additionally, we found that 46% of *M. chelonae* strains were susceptible to tobramycin (Table 3). *M. mageritense* isolates showed remarkably high resistance to clarithromycin and amikacin, but were susceptible to fluoroquinolones, imipenem, and cefoxitin (Table 3). The results of the antimicrobial susceptibility test and MICs for other rare RGM species are shown in Table 4 and Table S2, respectively. Amikacin and linezolid were the most effective against the 15 isolated RGM species (Table 4).

**Table 2:** Antimicrobial susceptibility of *Mycobacteroides abscessus* group (MAG) strains.

Antimicrobial agents	*M. abscessus* group (MAG: n = 313^A^)			subsp*. abscessus* (MAB: n = 178)			subsp. *massiliense* (MMA: n = 130)		
	Susceptibility^B)^ % (n)			Susceptibility % (n)			Susceptibility % (n)		
	S	I	R	S	I	R	S	I	R
Linezolid	33 (104)	19 (59)	48 (150)	30 (54)	19 (34)	51 (90)	37 (48)	19 (25)	44 (57)
Clarithromycin (ERT)	82 (256)	6 (20)	12 (37)	74 (131)	11 (19)	15 (28)	93 (121)	1 (1)	6 (8)
Clarithromycin (LRT)	43 (135)	3 (9)	54 (169)	11 (19)	3 (6)	86 (153)	89 (116)	2 (3)	9 (11)
Amikacin	87 (271)	12 (38)	1 (4)	90 (160)	9 (16)	1 (2)	82 (106)	17 (22)	1 (2)
Tobramycin	2 (7)	9 (27)	89 (279)	3 (5)	12 (22)	85 (151)	1 (2)	4 (5)	95 (123)
Imipenem	25 (80)	59 (184)	16 (49)	27 (48)	58 (103)	15 (27)	25 (32)	60 (78)	15 (20)
Ciprofloxacin	2 (6)	4 (12)	94 (295)	3 (5)	6 (10)	91 (163)	1 (1)	1 (2)	98 (127)
Moxifloxacin	5 (16)	6 (20)	89 (277)	6 (11)	9 (16)	85 (151)	4 (5)	3 (4)	93 (121)
Cefoxitin	23 (72)	69 (215)	8 (26)	25 (44)	67 (120)	8 (14)	22 (28)	69 (90)	9 (12)
minocycline	6 (18) ^C)^	8 (25) ^C)^	86 (269) ^C)^	2 (4)	3 (5)	95 (169)	11 (14)^C)^	16 (20) ^C)^	73 (95) ^C)^
ST^D)^	19 (58)		81 (255)	21 (37)		79 (141)	16 (21)		84 (109)

	MAG MIC (mg/L)			MAB MIC (mg/L)			MMA MIC (mg/L)		
	Range	MIC50	MIC90	Range	MIC50	MIC90	Range	MIC50	MIC90
Linezolid	< 1 to > 64	16	> 64	< 1 to > 64	32	> 64	< 1 to > 64	16	64
Clarithromycin (ERT)	< 0.12 to > 32	0.5	8	< 0.12 to > 32	1	32	< 0.12 to > 32	0.25	1
Clarithromycin (LRT)	< 0.12 to > 32	> 32	> 32	0.25 to > 32	> 32	> 32	< 0.12 to > 32	1	4
Azithromycin (ERT)	< 0.12 to > 32	4	> 32	0.25 to > 32	8	> 32	< 0.12 to > 32	1	4
Azithromycin (LRT)	< 0.25 to > 32	> 32	> 32	4 to > 32	> 32	> 32	< 0.25 to > 32	8	32
Arbekacin	< 1 to 64	8	16	< 1 to 64	8	16	< 1 to 32	8	16
Amikacin	< 1 to > 128	16	32	< 1 to > 128	16	32	< 1 to 64	16	32
Gentamycin	< 1 to > 128	16	32	< 1 to > 128	16	32	< 1 to 64	16	32
Tobramycin	1 to > 64	16	32	1 to > 64	16	32	2 to > 64	16	64
Imipenem	< 0.5 to > 64	8	32	< 0.5 to > 64	8	32	2 to > 64	8	32
Doripenem	2 to > 128	64	> 128	2 to > 128	64	128	8 to > 128	64	> 128
Faropenem	< 1 to > 128	128	> 128	< 1 to > 128	128	> 128	8 to > 128	128	> 128
Levofloxacin	< 0.5 to > 32	32	> 32	< 0.5 to > 32	32	> 32	4 to > 32	32	> 32
Sitafloxacin	0.12 to > 4	2	> 4	0.12 to > 4	2	> 4	0.25 to > 4	2	> 4
Ciprofloxacin	0.25 to > 16	16	> 16	0.25 to > 16	16	> 16	1 to > 16	16	> 16
Moxifloxacin	0.12 to > 8	8	> 8	0.12 to > 8	8	> 8	1 to > 8	> 8	> 8
Cefmetazole	< 1 to > 128	32	128	< 1 to > 128	32	128	8 to > 128	32	128
Cefoxitin	4 to > 64	32	64	4 to > 64	32	64	8 to > 64	32	64
Ceftriaxone	8 to > 64	> 64	> 64	8 to > 64	> 64	> 64	16 to > 64	> 64	> 64
Cefepime	4 to > 32	> 32	> 32	8 to > 32	> 32	> 32	32—> 32	> 32	> 32
Ethanbutol	8 to > 128	64	128	8 to > 128	64	128	8—> 128	64	128
Rifabutin	< 0.125 to > 8	8	> 8	< 0.125 to > 8	4	> 8	1—> 8	8	> 8
Minocycline	< 0.5 to > 32	> 32	> 32	< 0.5 to > 32	> 32	> 32	< 0.5 to > 32	16	> 32
Tigecycline	0.25 to > 8	1	4	0.25 to > 8	1	4	0.25 to > 8	2	8
AMPC/CVA^E)^	8/4 to > 128/64	> 128/64	> 128/64	8/4 to > 128/64	> 128/64	> 128/64	8/4 to > 128/64	> 128/64	> 128/64
ST	< 0.25/4.8 to > 8/152	8/152	> 8/152	< 0.25/4.8 to > 8/152	8/152	> 8/152	0.5/9.5 to > 8/152	8/152	> 8/152

A) These strains included 5 M*. abscessus* subsp. *bolletii* (MBO).

B) S: Susceptible, I: intermediate, R: resistant.

C) Minimum inhibitory concentration (MIC) data for minocycline could not be determined for one strain.

D) Trimethoprim/sulfamethoxazole.

E) Amoxicillin/clavulanic acid.

**Table 3:** Antimicrobial susceptibility of major rapidly growing mycobacteria (RGM) strains other than *M. abscessus* group (MAG).

Antimicrobial agents	*M. fortuitum*			*M. chelonae*			*M. peregrinum*			*M. mageritense*		
	**(n = 85)**			**(n = 55)**			**(n = 11)**			**(n = 10)**		
	Susceptibility %			Susceptibility %			Susceptibility %			Susceptibility %		
	S	I	R	S	I	R	S	I	R	S	I	R
Linezolid	71	14	15	49	33	18	82	18	0	80	10	10
Clarithromycin (ERT)	18	12	70	96	4	0	82	0	18	0	0	100
Clarithromycin (LRT)	0	1	99	89	4	7	82	0	18	0	0	100
Amikacin	100	0	0	62	34	4	100	0	0	0	60	40
Tobramycin	4	0	96	46	47	7	0	18	82	0	0	100
Imipenem	94	5	1	18	53	29	82	18	0	70	20	10
Ciprofloxacin	95	1	4	5	20	75	91	0	9	89^A)^	11 ^A)^	0 ^A)^
Moxifloxacin	97	2	1	7	20	73	91	0	9	89 ^A)^	11 ^A)^	0 ^A)^
cefoxItin	15	80	5	5	5	90	100	0	0	80	20	0
Minocycline	41	7	52	16	4	80	18	9	73	0	60	40
ST^B)^	88		12	24		76	100		0	40		60

	**MIC (mg/L)**			**MIC (mg/L)**			**MIC (mg/L)**			**MIC (mg/L)**		
	Range	MIC50	MIC90	Range	MIC50	MIC90	Range	MIC50	MIC90	Range	MIC50	MIC90
Linezolid	< 1 to > 64	4	32	< 1 to > 64	16	32	< 1 to 16	4	16	1 to 32	4	16
Clarithromycin (ERT)	< 0.12 to 4	8	> 32	< 0.12 to > 32	0.25	1	< 0.12 to 16	1	8	32 to > 32	> 32	> 32
Clarithromycin (LRT)	< 0.12 to > 32	> 32	> 32	< 0.12 to > 32	1	4	< 0.12 to > 32	1	> 32	> 32	> 32	> 32
Azithromycin (ERT)	0.5 to > 32	> 32	> 32	< 0.12 to 16	2	8	0.25 to > 32	2	> 32	> 32	> 32	> 32
Azithromycin (LRT)	16 to > 32	> 32	> 32	0.25 to > 32	8	32	0.5 to > 32	16	> 32	> 32	> 32	> 32
Arbekacin	< 1 to 16	4	4	< 1 to 16	4	8	< 1 to 4	< 1	4	8 to 64	32	64
Amikacin	< 1 to 16	2	4	2 to 64	16	32	< 1 to 8	< 1	2	32 to > 128	64	128
Gentamycin	< 1 to 128	8	16	2 to 32	8	16	< 1 to 8	4	8	16 to > 128	64	128
Tobramycin	2 to > 64	32	64	< 0.5 to 32	4	4	4 to 16	8	8	64 to > 64	> 64	> 64
Imipenem	1 to > 64	4	4	1 to > 64	16	32	1 to 8	4	8	2 to 32	2	16
Doripenem	2 to 128	8	16	4 to > 128	> 128	> 128	2 to 16	8	16	2 to 64	4	32
Faropenem	2 to > 128	16	32	32 to > 128	> 128	> 128	< 1 to 32	8	16	2 to 128	4	32
Levofloxacin	< 0.5 to 32	< 0.5	2	2 to > 32	16	> 32	< 0.5 to 32	< 0.5	4	16 to > 32	< 0.5	2
Sitafloxacin	< 0.03 to 1	0.12	0.25	0.25 to > 4	1	4	< 0.03 to 1	0.06	0.12	< 0.03 to 0.5	0.06	0.25
Ciprofloxacin	< 0.12 to > 16	0.25	1	1 to > 16	4	> 16	< 0.12 to > 16	< 0.12	0.5	0.25 to 2	0.25	2
Moxifloxacin	< 0.06 to > 8	0.12	0.5	0.5 to > 8	4	> 8	< 0.06 to 4	0.12	0.25	0.12 to 2	0.12	2
Cefmetazole	4 to > 128	16	32	16 to > 128	> 128	> 128	4 to 64	4	8	4 to 16	8	16
Cefoxitin	2 to > 64	32	64	16 to > 64	> 64	> 64	8 to 16	8	16	16 to 32	16	32
Ceftriaxone	4 to > 64	> 64	> 64	8 to > 64	> 64	> 64	> 64	> 64	> 64	> 64	> 64	> 64
Cefepime	8 to > 64	> 32	> 32	8 to > 64	> 32	> 32	> 32	> 32	> 32	> 32	> 32	> 32
Ethanbutol	2 to > 128	16	64	32 to > 128	128	> 128	2 to > 128	16	64	16 to 128	32	64
Rifabutin	0.25 to > 8	2	8	1 to > 8	8	> 8	0.5 to 8	2	4	2 to > 8	8	> 8
Minocycline	< 0.5 to > 32	8	> 32	< 0.5 to > 32	32	> 32	< 0.5 to 16	8	> 32	2 to 32	4	8
Tigecycline	0.12 to 4	0.5	1	0.12 to 4	1	2	0.12 to 4	0.5	1	0.12 to 1	0.25	1
AMPC/CVA^C)^	2/1 to > 128/64	32/16	128/64	32/16 to > 128/64	> 128/64	> 128/64	8/4 to > 128/64	128/64	> 128/64	8/4 to 128/64	16/8	64/32
ST^B)^	< 0.25/4.8 to > 8/152	1/19	4/76	< 0.5/9.5 to > 8/152	4/76	> 152/8	< 0.25/4.8 to 1/19	< 0.25/4.8	1/19	1/19 to > 8/152	4/76	> 8/152

A) Minimum inhibitory concentration (MIC) for ciprofloxacin and moxifloxacin could not be determined for one strain.

B) Trimethoprim/sulfamethoxazole.

C) Amoxicillin/clavulanic acid.

**Table 4:** Characteristics of antimicrobial susceptibility of rapidly growing mycobacteria (RGM) species.

	Linezolid	Clarithromycin	Amikacin	Tobramycin	Imipenem	Cefoxitin	Moxifloxacin	Minocycline	ST^A)^
MAB	△	▲	◎	▲	△	△	▲	▲	▲
MMA	△	◎	◎	▲	△	▲	▲	▲	▲
MBO	△	▲	◎	▲	▲	▲	▲	▲	▲
*M. fortuitum*	〇	▲	◎	▲	◎	▲	◎	△	◎
*M. chelonae*	△	◎	〇	△	▲	▲	▲	▲	▲
*M. peregrinum*	◎	◎	◎	▲	◎	◎	◎	▲	◎
*M. mageritense*	◎	▲	▲	▲	〇	◎	◎	▲	△
*M. septicum*	◎	▲	◎	▲	◎	△	◎	▲	◎
*M. mucogenicum*	◎	〇	◎	▲	◎	◎	〇	▲	◎
*M. porcinum*	〇	▲	◎	▲	◎	〇	◎	▲	◎
*M. wolinskyi*	◎	▲	◎	▲	▲	▲	◎	◎	▲
*M. senegalense*	〇	〇	◎	▲	〇	〇	◎	〇	〇
*M. goodie*	◎	▲	◎	▲	◎	〇	◎	〇	◎
*M. iranicum*	◎	◎	◎	▲	◎	◎	◎	◎	◎
*M. canariasense*	◎	▲	◎	◎	▲	◎	◎	▲	◎
*M. immunogenum*	◎	〇	▲	▲	▲	▲	▲	▲	◎
*M. sphagni*	◎	▲	◎	◎	〇	▲	◎	▲	◎

◎Susceptible isolates > 75%, 〇 Susceptible isolates 51%‒75%, △Susceptible isolates 25%‒50%, ▲Susceptible isolates < 25%

A) Trimethoprim/sulfamethoxazole.

We also investigated the MICs against RGM for antibacterial drugs for which CLSI did not set breakpoints. The MIC50 of sitafloxacin was the lowest among all the fluoroquinolones for all RGM species (Tables 2, 3). Except in *M. fortuitum* and *M. wolinskyi* isolates, the MIC50 of arbekacin was the lowest among aminoglycoside antimicrobials (Tables 2, 3). The MIC50 of cefmetazole was equal to or lower than that of cefoxitin for all RGM species, although the values were similar (Tables 2, 3). The MIC50 of rifabutin was lower among MAB than among MMA. The proportion of isolates with MIC ≤ 2 mg/L for rifabutin was significantly higher than that of MMA isolates [MAB: 50/178 (28.1%) vs. MMA: 23/130 (17.7%); *p* = 0.041] (Table 2). Faropenem had a higher MIC50 than imipenem among all RGM isolates except *M. iranicum* (Tables 2, 3).

### Relationship between MAB *erm*(41) sequevar type and susceptibility to clarithromycin

The sequence of *erm*(41) was obtained from 180 isolates, and the relationship between MAB *erm*(41) sequevar and clarithromycin MIC was determined (Table 5). For the remaining three isolates, we could not obtain any sequence data. None of the MAB isolates had a truncated *erm*(41) sequevar, whereas 2 of 133 MMA isolates had a functional *erm*(41) T28 sequevar. The clarithromycin (late-reading-time [LRT]) MICs for these two isolates were 0.5 mg/L and 8 mg/L, respectively. The *erm*(41) gene sequences of 131 MMA isolates were identical. In this survey, the proportion of the C28 sequevar in MAB was 12.2% (22/180), all of which were type 2. Several new sequevar types were identified in our isolates; the two most common of these new isolates were named jpn1 and jpn2. These new sequevars were similar to type 10, and all isolates were resistant to clarithromycin. The single nucleotide polymorphisms of *erm*(41) in each sequevar are shown in Table S3. Of the 158 isolates of the T28 sequevar, 7 showed clarithromycin MIC ≤ 4 mg/L, including isolates of types 1, 6, 7, 8, and 10.

**Table 5:** *erm*(41) sequevar type and clarithromycin minimum inhibitory concentration (MIC) of *M. abscessus* subsp. *abscessus* (MAB).

Sequevar type	Distribution (no. of strains) of MIC (mg/L) of clarithromycin											
	≦0.12	0.25	1	1	2	4	8	16	32	32 <	not growing^A)^	total
Type1			1		1	1				45	1	49
Type2		1	1	7	4	5		2	1	1		22
Type6					1				1	20	2	24
Type7					1					27	1	29
Type8					1					2		3
Type9										4		4
Type10					1				1	10		12
jpn1										24		24
jpn2										4		4
Other^B)^							1			7	1	9
Unknown^C)^										3		3
Total		1	2	7	9	6	1	2	3	147	5	183

A) These strains did not grow sufficiently in the control well, and MIC could not be determined.

B) Other rare sequevar types. These types did not include types 3, 4, and 5.

C) PCR was performed to obtain a product, but sufficient *erm*(41) gene sequence data could not be obtained.

### Relationship between *rrl* gene mutation of MAG and susceptibility to clarithromycin

Among the 37 MAG isolates with acquired macrolide resistance, the proportions with *rrl* mutations were 2/24 for the MAB T28 sequevar, 0/2 for the MAB C28 sequevar, 1/2 for MAB unknown, 1/1 for MBO, and 3/8 for MMA (Table 6). However, the rate of *rrl* mutation among MMA isolates that acquired macrolide resistance was higher than that of the MAB T28 sequevar, although not significantly (MAB T28 sequevar: 2/24 [8.3%] vs. MMA: 3/8 [37.5%]; *p* = 0.085).

**Table 6:** Frequency of *rrl* mutation in 37 *Mycobacteroides abscessus* group (MAG) strains.

MAG subspecies (n)	MIC (mg/L)^A)^ of clarithromycin			
	8	16	32	32 <
**MAB T28 (24)**				
WT	3	3	4	10
A2058C				2
unknown^B)^		1	1	
**MAB C28 (2)**				
WT		1	1	
**MAB unknown**^**C)**^** (2)**				
WT				1
A2057G	1			
**MMA (8)**				
WT	1	1		3
A2058C				1
A2058G				2
**MBO (1)**				
A2059G				1
**total**	5	6	6	20

A) Acquired resistance = CAM minimum inhibitory concentration (MIC) ≥ 8 mg/L at early-reading-time.

B) PCR was performed to obtain a product, but sufficient *rrl* gene sequence data could not be obtained.

C) PCR was performed to obtain a product, but sufficient *erm*(41) gene sequence data could not be obtained.

### Relationship between *rrs* gene mutation and susceptibility to amikacin

*rrs* (A1408G) mutations were not found among the 73-amikacin non-susceptible (MIC ≥ 32 mg/L) isolates (MAG, *M. chelonae*, and *M. mageritense*).

### Quinolone resistance of *M. fortuitum* and its mechanism

Of the three isolates of *M. fortuitum* that were resistant to ciprofloxacin, only one had a mutation in *gyrA*. In the mutant strain, the *gyrA* gene resulted in S83W amino acid substitution (TCG → TGG). None of the ciprofloxacin-susceptible isolates had mutations in *gyrA* and *gyrB*.

## Discussion

In this study, we accurately identified 15 species of RGM from clinical isolates obtained from different locations around Japan. We characterized the susceptibility of these isolates to 24 antimicrobials, including tigecycline, sitafloxacin, rifabutin, and cefmetazole; none have defined MIC breakpoints in the CLSI, but they may have potential as therapeutic agents for RGM infections. We investigated not only MAG antimicrobial susceptibility, but also several gene mutations involved in antimicrobial resistance and prepared a summary of the susceptibility of the remaining 14 species of RGM.

The proportion of the C28 sequevar in MAB isolated from lower respiratory specimens (LRS) has been reported to be approximately 16‒35%^4, 10, 16, 17^. However, in some previous Japanese reports, the ratio of the C28 sequevar among MAB from LRS was very low at 4.2% (2/48)^18^. In our survey, it was 12.2% (22/180), which is higher than that in the previous report^18^. In Japan, it is necessary to continue to evaluate whether the proportion of the C28 sequevar in MAB is lower than those in other countries.

A previous report from the USA indicated that sequevar types 4, 6, 7, 8, 9, and 10 (all T28 sequevars) may be associated with macrolide-induced resistance^10^. However, similar assessments outside of the USA have not been conducted so far. Among the 180 MABs in our study, only 4 isolates with *erm*(41) sequevar types 6, 7, 8, and 10 were susceptible to clarithromycin. Our data were generally consistent with the previous report^10^. Therefore, it was suggested that these sequevars are macrolide-resistant. So far, CLSI has recommended the determination of the *erm*(41) sequevar type for evaluation of induced macrolide resistance in MAB^11^, and our results support this recommendation. Further investigations on the relationship between sequevar types and macrolide resistance in other regions are required.

The *rrl* gene mutation is more likely to occur in the MAB C28 sequevar and MMA than in the MAB T28 sequevar among clarithromycin-acquired resistant strains in MAG^4^. In our survey, we found a similar trend but could not show a significant difference. Among MAG, more than half of the macrolide-acquired resistance occurred by mechanisms other than *rrl* gene mutation. The exact mechanism remains to be investigated.

Additionally, none of the amikacin non-susceptible isolates in our survey had the *rrs* gene mutation. A previous French study of antimicrobial susceptibility in 165 isolates of MAG showed that 7/8 strains with amikacin MIC > 64 mg/L had a *rrs* A1408G gene mutation^16^, which suggested that amikacin MIC > 64 mg/L is a criterion to suspect amikacin-acquired resistance^16^. In our survey, only one isolate of MAG showed MIC > 64 mg/L, and none of the isolates showed *rrs* mutation. MAG isolated in Japan may have fewer amikacin-acquired resistant isolates than those isolated in France.

As reported previously^18, 19^, *M. fortuitum* was resistant to clarithromycin; however, it was susceptible to aminoglycosides, carbapenems, and fluoroquinolones in our study. Previous reports suggest that, in *M. fortuitum*, a serine residue at the 83^rd^ position of *gyrA* constitutes QRDR and contributes to susceptibility to fluoroquinolones compared with other NTMs^6^. However, to date, only one report has shown quinolone resistance due to mutations in *gyrA*^19^. There has been no report of mutations in a serine residue at the 83^rd^ position of *gyrA*. Fluoroquinolone resistance was found in 3 of 85 (3.5%) isolates in our study, and the S83W amino acid substitution was present in one of the three isolates. Our result also suggests that fluoroquinolone resistance can occur based on genetic changes other than QRDR mutations, and it is necessary to clarify the resistance mechanism in the future. In Japan, fluoroquinolones are being overused^20^, and there is a concern regarding the increase of fluoroquinolone-resistant isolates in *M. fortuitum*. Because *M. fortuitum* shows induced resistance to macrolides, fluoroquinolones play an important role in the treatment of *M. fortuitum* infections as an oral antibiotic. There is a great concern regarding treatment efficacy with the increase in resistant isolates.

Among *M. chelonae* isolates, resistance to clarithromycin was found in approximately 10% of isolates, consistent with previous reports^18, 21^. Previous reports seem to indicate regional variability in tobramycin susceptibility, ranging from 54% in the UK^21^ to 83% and 17% in Japan^18, 22^. In our study, approximately 40% of the strains were tobramycin-susceptible, an intermediate value between the values reported by the two previous reports from Japan. In addition, no *rrs* mutations were found in amikacin non-susceptible isolates. Arbekacin may be a potential therapeutic for isolates that are less susceptible to amikacin and tobramycin.

*M. peregrinum* was susceptible to most of the tested antimicrobials. *M. mageritense* isolates were resistant to clarithromycin, as has been previously reported^23^, and showed a low susceptibility to amikacin, although none had a *rrs* gene mutation (3 isolates showed an amikacin MIC > 64 mg/L). The mechanism of *M. mageritense* resistance to amikacin remains to be investigated. Conversely, it showed good susceptibility to quinolones, cefoxitin, and linezolid.

There are few reports on antimicrobial susceptibility for other rare RGM species using a sufficiently high number of clinical isolates. There is only one study involving *M. mucogenicum* and *M. immunogenum* reporting that most of the isolates were susceptible to linezolid, amikacin, and trimethoprim/sulfamethoxazole, while showing a poor susceptibility to clarithromycin^24^. In our study, although the number of isolates was small, we could show the tendency of antimicrobial susceptibility for rare RGM species. These rare RGM species tended to be susceptible to linezolid, quinolones, and trimethoprim/sulfamethoxazole.

Although there have been no reports regarding the MIC of arbekacin in RGM, this antimicrobial showed the lowest MIC among the aminoglycosides for almost all RGM species in this study (Tables 2, 3). The MIC50 value of sitafloxacin is reported to be lower than that of other fluoroquinolones in MAG, *M. fortuitum*, and *M. chelonae*^18^. However, in this study, we showed that the effect of sitafloxacin was similar on the 15 RGM species (Tables 2, 3). Cefmetazole and cefoxitin, cephamycin-based antimicrobials, had similar MICs, consistent with previous reports^25, 26^ (Tables 2, 3). In countries such as Japan, when patients cannot be administered cefoxitin, cefmetazole may be an option for RGM treatment. In recent years, rifabutin has attracted attention as an oral treatment for MAG^27, 28^, but, so far, there have been few reports of MICs measured by micro-dilution using cation-adjusted Mueller–Hinton broth medium^28^. Here, we have not only measured rifabutin MICs for many isolates using this standard method, but also showed that MICs were lower for MAB than for MMA (Table 2). MAB has a very high resistance rate not only to clarithromycin but also to fluoroquinolone; thus, finding an alternative orally administrated therapeutic option is essential. A detailed evaluation is required in the future to determine whether rifabutin will be an effective orally administered therapeutic option.

There are some limitations to our study. It was unclear whether there was prior administration of antibacterial drugs before susceptibility testing for all isolates. Some of the RGM species isolated in this study were rarely isolated to evaluate drug susceptibility. However, despite these limitations, our study reveals important epidemiological information about RGM in Japan and suggests several drugs that can be investigated as new treatment candidates. It is therefore necessary to accumulate and evaluate data from a larger set of samples and to verify the correlation between the actual therapeutic effect and the MIC values of these drugs in clinical trials.

We showed antimicrobial susceptibility profiles of 15 RGM species isolated in Japan. Amikacin and linezolid were the most effective against the 15 isolated RGM species. Arbekacin, sitafloxacin, and cefmetazole may be possible therapeutic options for RGM infections. Based on the MIC values, rifabutin may be more potent in the treatment of MAB than MMA. Clinical trials are needed in the future to validate our findings.

## Methods

### Clinical isolates

From January 2012 to March 2019, 509 clinical specimens [409 LRS, 87 non-lower respiratory specimens, and 13 unknown] isolated from patients in Japan (one specimen per patient) were included in this study. From the specimens, 403 strains were isolated at BioMedical Laboratories (BML), Inc., a major clinical laboratory, and 106 were isolated at 45 hospitals in Japan.

### PCR and sequence analysis

Bacterial genomic DNA was extracted using ISOPLANT II (NIPPON GENE CO., LTD, Japan). The three housekeeping genes, *hsp65*^29^, *rpoB*^30^*,* and *sodA*^31^, of each isolate were sequenced for RGM species identification. For MAG, an additional PCR-based typing scheme^32^ was used for subspecies identification, and the *erm*(41) sequence type was determined^9, 10^. The sequence of *rrl* from MAG strains exhibiting acquired macrolide resistance was also determined^33^. Similarly, the sequences of the *rrs* gene from MAG, *M. chelonae*, and *M. mageritense,* which are amikacin non-susceptible isolates, were analyzed^5^. The sequences of the *gyrA* and *gyrB* genes encoding the QRDR were elucidated for all *M. fortuitum* isolates. All PCR procedures were performed as described previously^5, 9, 29–33^, and the primers used are shown in Table S1.

### Antimicrobial susceptibility test

All strains were subcultured on trypticase soy agar with 5% sheep blood (Becton, Dickinson and Company, New Jersey) at 35 °C for 3‒5 days. Antimicrobial susceptibility testing was performed per the recommendations in the CLSI M24A-2 at 30 °C^8^. A nephelometer (VITEK DENSICHEK, bioMerieux, France) was used to standardize the inoculum density (0.5 McFarland standard). The MICs of 24 antimicrobial agents (tigecycline, linezolid, clarithromycin, azithromycin, arbekacin, amikacin, gentamycin, tobramycin, imipenem, doripenem, faropenem, levofloxacin, sitafloxacin, ciprofloxacin, moxifloxacin, cefmetazole, cefoxitin, ceftriaxone, cefepime, ethambutol, rifabutin, minocycline, amoxicillin/clavulanic acid, and trimethoprim/sulfamethoxazole) were measured by the micro-dilution method using cation-adjusted Mueller–Hinton broth medium (Becton, Dickinson and Company)^8^. The MICs of clarithromycin and azithromycin were read two times to detect induced resistance. Positive growth of the control between days 3 and 5 was defined as early-reading-time. Inducible macrolide resistance was determined on day 14 and defined as LRT. Repetition of MIC measurement, as recommended by guidelines, was performed without exception.

### Statistical analysis

Statistical analyses were performed using GraphPad Prism ver. 8.2.0 for Windows (GraphPad Software, San Diego, CA, USA). Data were compared using the Chi-square test for categorical variables, whereas Fisher’s exact test was used where the assumption of the Chi-square test was violated.

## Supplementary Information

Supplementary Information.

## Data Availability

The dataset generated and analyzed during the current study is available from the corresponding author on reasonable request.
